# Supplementary figures and images for: LRRC15 inhibits SARS-CoV-2 cellular entry in trans
Source: PLoS Biol. 2022 Oct 13;20(10):e3001805. doi: 10.1371/journal.pbio.3001805 (PMC9595563; doi:10.1371/journal.pbio.3001805)

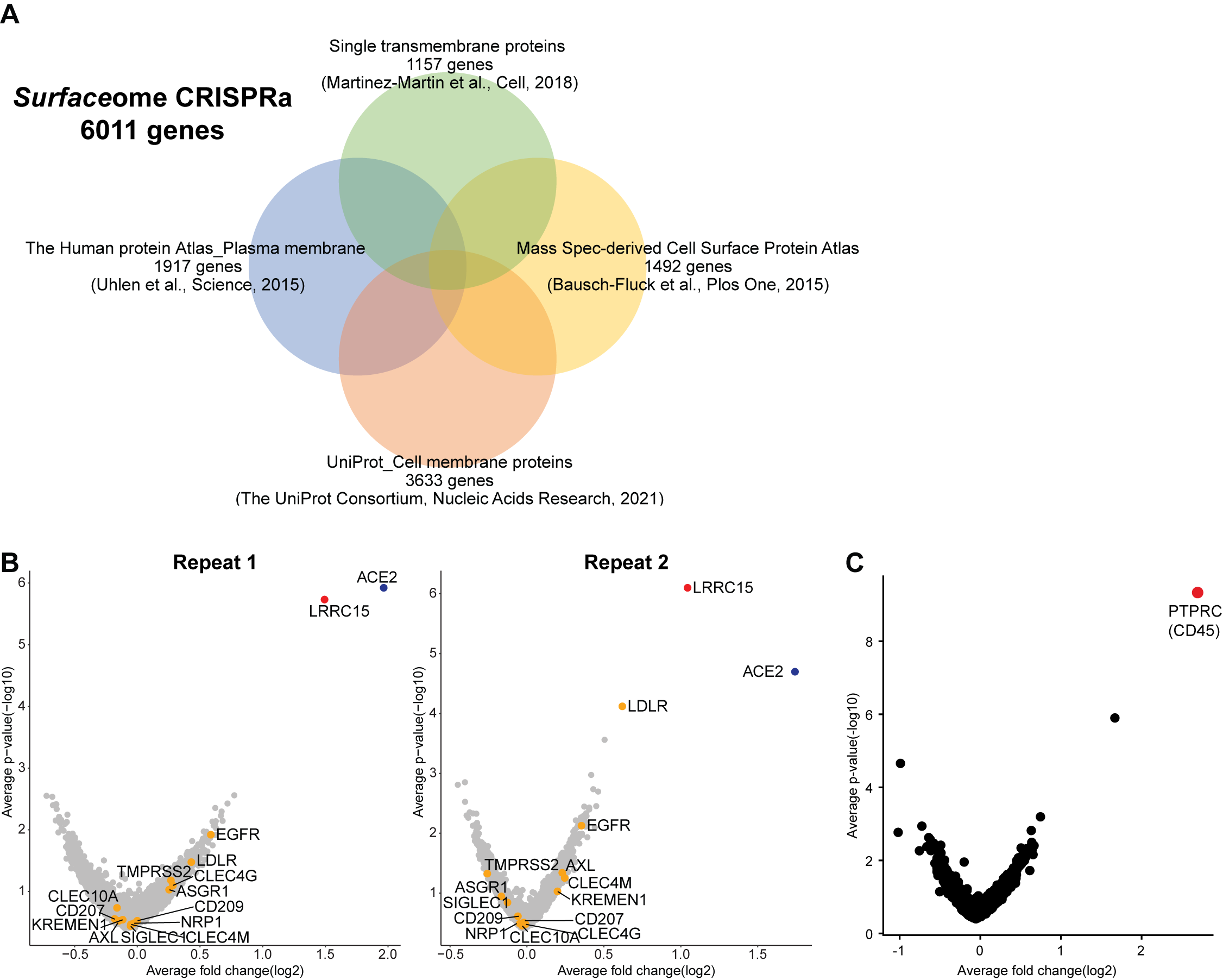

Supplement: S1 Fig — (A) Venn diagram shows the composition of the surfaceome CRISPRa library, which was made by integrating 4 datasets for plasma membrane proteins [59–62]. (B) Volcano plot showing sgRNAs targeting spike attachment factors in cells binding with SARS-CoV-2 spike S1-Fc. (C) Volcano plot showing sgRNAs enriched or depleted in cells sorted after staining with anti-CD45. For underlying data, see S1 Data. CRISPRa, CRISPR activation; SARS-CoV-2, Severe Acute Respiratory Syndrome Coronavirus 2; sgRNA, single guide RNA. (TIF) [file pbio.3001805.s004.tif]

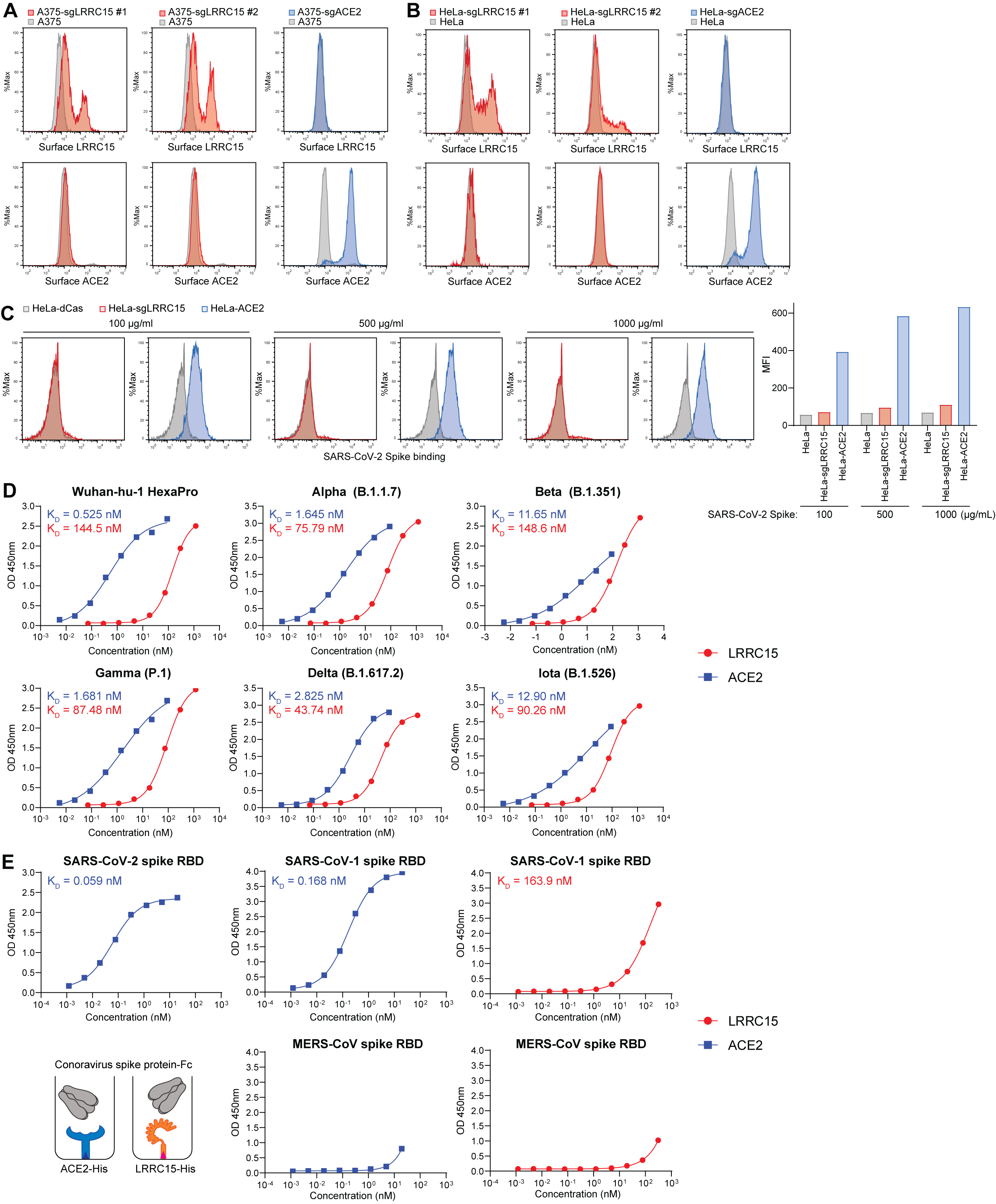

Supplement: S2 Fig — (A, B) A375 cells (A) or HeLa cells (B) were transduced with indicated activating sgRNAs and surface expression of LRRC15 and ACE2 was measured by flow cytometry. (C) HeLa cells were transduced with indicated activating sgRNAs and incubated with 100, 500, or 1,000 μg/mL full-length SARS-CoV-2 spike protein. Protein binding was measured by flow cytometry and calculated as MFI. (D) The binding of ACE2 or LRRC15 to immobilized histidine-tagged spike proteins from SARS-CoV-2 mutant variants was measured using ELISA assay. (E) The binding affinity of ACE2 or LRRC15 to SARS-CoV-2, SARS-CoV-1, and MERS-CoV spike protein RBD fragment was determined by ELISA. For underlying data, see S3 Data. ACE2, angiotensin-converting enzyme 2; LRRC15, leucin-rich repeat-containing 15; MERS-CoV, Middle East Respiratory Syndrome Coronavirus; MFI, mean fluorescence intensity; RBD, receptor-binding domain; SARS-CoV-1, Severe Acute Respiratory Syndrome Coronavirus; SARS-CoV-2, Severe Acute Respiratory Syndrome Coronavirus 2; sgRNA, single guide RNA. (TIF) [file pbio.3001805.s005.tif]

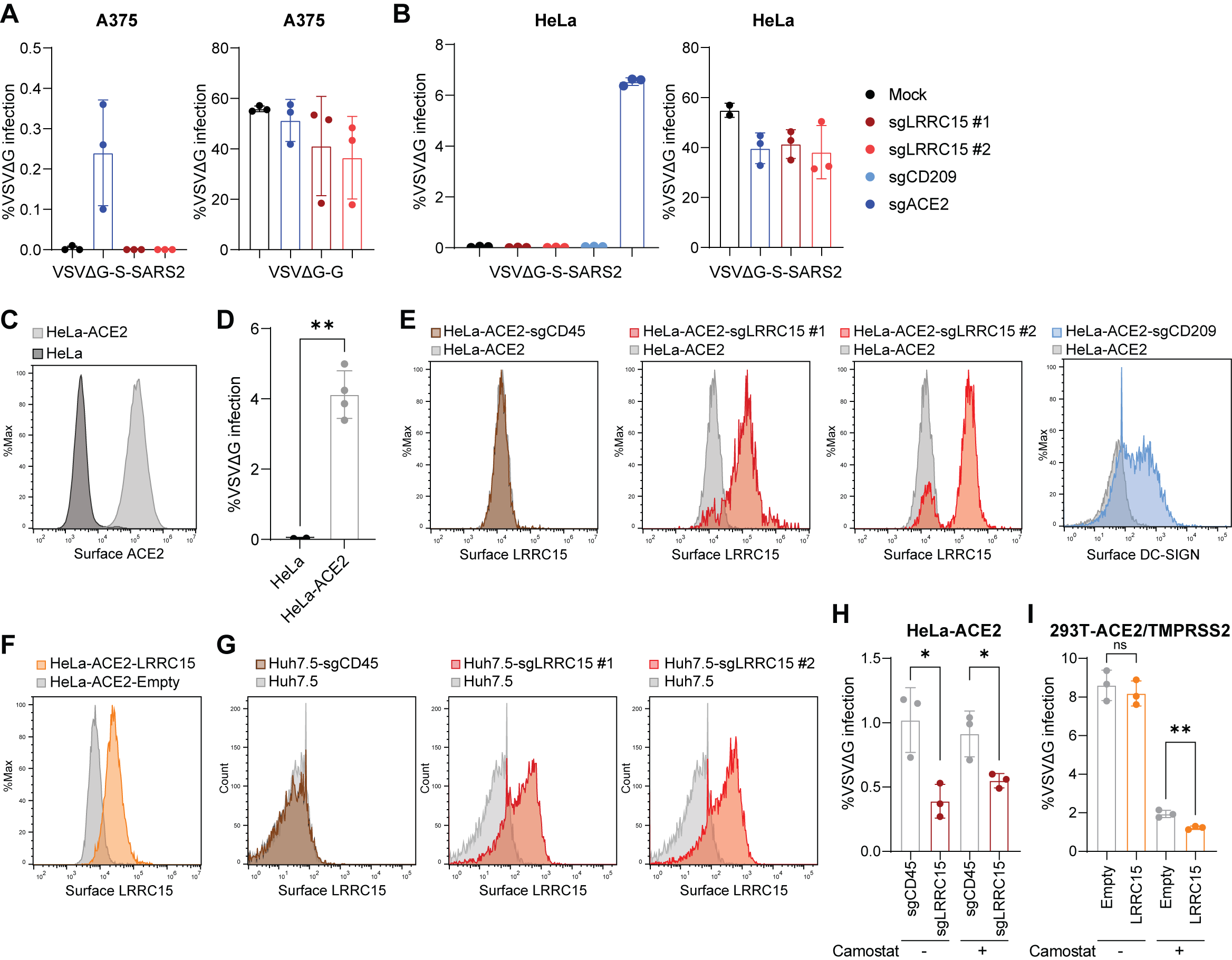

Supplement: S3 Fig — (A, B) A375 cells (A) or HeLa cells (B) transduced with indicated activating sgRNAs were infected with VSV pseudoviruses VSVΔG-S-SARS2 or VSVΔG-G. GFP signal was measured at 20 hpi by flow cytometry (n = 3). (C) HeLa cells stably expressing ACE2 (HeLa-ACE2) were assessed for cell surface expression of ACE2 by flow cytometry. (D) HeLa or HeLa-ACE2 cells were infected with VSV pseudovirus harboring SARS-CoV-2 spike and GFP signal was measured at 20 hpi by flow cytometry (n = 4). (E) HeLa-ACE2 cells transduced with indicated activating sgRNAs were assessed for surface expression of LRRC15 by flow cytometry. (F) HeLa-ACE2 cells expressing LRRC15 or empty vector were assessed for surface expression of LRRC15 by flow cytometry. (G) Huh7.5 cells transduced with indicated activating sgRNAs were assessed for surface expression of LRRC15 by flow cytometry. (H, I) HeLa-ACE2 cells transduced with indicated activating sgRNAs (H) or 293T-ACE2/TMPRSS2 cells expressing LRRC15 or empty vector (I) were pretreated with DMSO or camostat for 1 h and infected with VSVΔG-S-SARS2. GFP signal was measured at 20 hpi by flow cytometry (n = 3). Data represent means ± SD (A, B, D, H, I). Data were analyzed unpaired two-tailed t test (D, H, I); ns, not significant; *p < 0.05; **p < 0.01. For underlying data, see S3 Data. ACE2, angiotensin-converting enzyme 2; GFP, green fluorescent protein; hpi, hours postinfection; LRRC15, leucin-rich repeat-containing 15; SARS-CoV-2, Severe Acute Respiratory Syndrome Coronavirus 2; sgRNA, single guide RNA; TMPRSS2, transmembrane protease serine 2; VSV, vesicular stomatitis virus. (TIF) [file pbio.3001805.s006.tif]

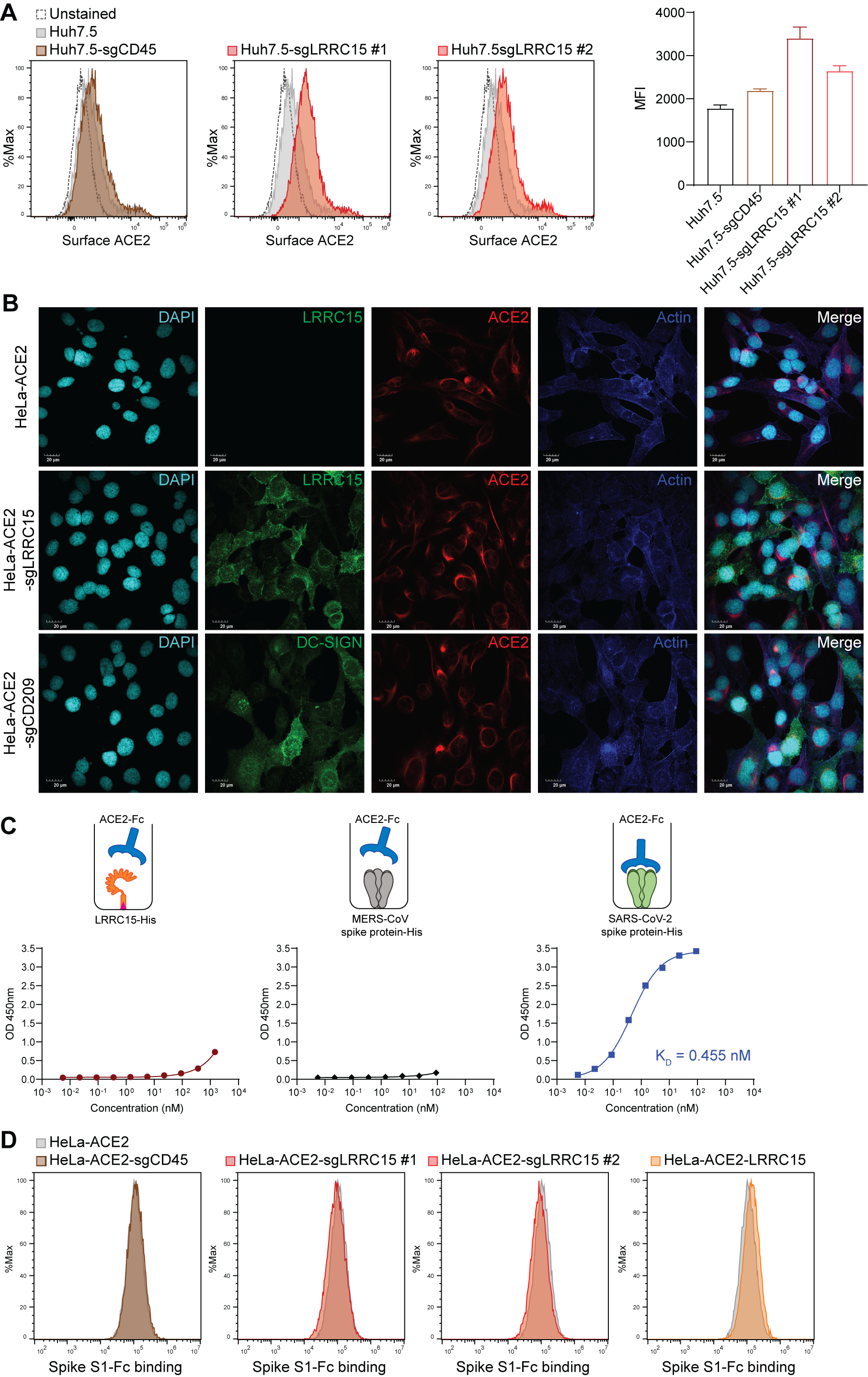

Supplement: S4 Fig — (A) Huh7.5 cells were transduced with indicated activating sgRNAs. Cell surface expression of ACE2 was measured by flow cytometry and calculated as MFI. Data represent means ± SD (n = 3). (B) Representative images of immunofluorescence staining of LRRC15 or DC-SIGN (green), ACE2 (red), Actin (blue), and DAPI (cyan) in HeLa-ACE2 cells transduced with indicated activating sgRNAs. (C) Binding of Fc-tagged recombinant human ACE2 to His-tagged LRRC15 was measured by ELISA. SARS-CoV-2 spike protein was used as a positive control, MERS-spike protein as a negative control. (D) Binding of SARS-CoV-2 spike S1-Fc to HeLa-ACE2 cells transduced with indicated activating sgRNAs or a LRRC15-expressing vector was measured by flow cytometry. For underlying data, see S3 Data. ACE2, angiotensin-converting enzyme 2; LRRC15, leucin-rich repeat-containing 15; MERS, Middle East Respiratory Syndrome; MFI, mean fluorescence intensity; SARS-CoV-2, Severe Acute Respiratory Syndrome Coronavirus 2; sgRNA, single guide RNA. (TIF) [file pbio.3001805.s007.tif]

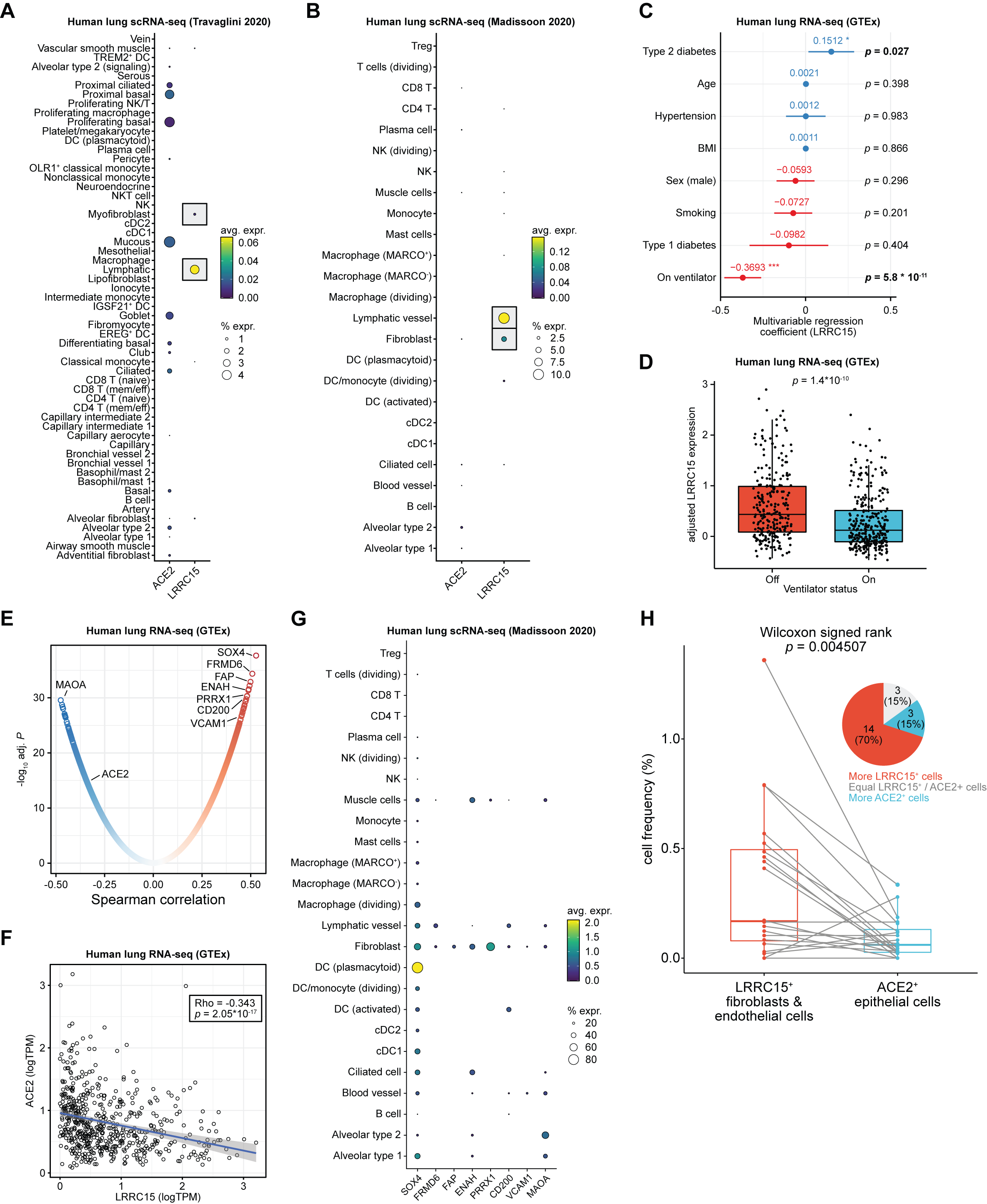

Supplement: S5 Fig — (A, B) Cell type–specific expression of ACE2 and LRRC15, assessed by scRNA-seq of human lungs from (A) the Human Lung Cell Atlas [41] or (B) the Tissue Stability Cell Atlas [42]. (C) Forest plot of the multivariable regression coefficients for various clinical features as predictors for lung expression of LRRC15 (from GTEx; n = 554 samples) [43]. (D) Tukey boxplots (IQR boxes with 1.5 × IQR whiskers) showing LRRC15 expression after adjustment for clinical features noted in (C), classifying each lung sample by ventilator status at the time of death. Statistical significance was assessed by two-tailed unpaired Mann–Whitney test. (E) Spearman correlation analysis between LRRC15 and all other genes, based on RNA-seq of human lung samples from GTEx. Multiple hypothesis correction was performed by the Benjamini–Hochberg method. (F) Scatter plot comparing log-normalized expression of LRRC15 and ACE2 across human lung samples. Spearman rho = −0.343, p = 2.05 * 10−17. The linear regression line is overlaid, with 95% confidence intervals shaded in. (G) Cell type–specific expression of genes that are highly correlated with LRRC15, assessed by scRNA-seq of human lungs from the Tissue Stability Cell Atlas. (H) Frequency of LRRC15-expressing fibroblasts and endothelial cells and ACE2-expressing epithelial cells were assessed by scRNA-seq of human lungs and presented in Tukey boxplots (IQR boxes with 1.5 × IQR whiskers). For underlying data, see S2 Data. ACE2, angiotensin-converting enzyme 2; GTEx, Genotype-Tissue Expression; IQR, interquartile range; LRRC15, leucin-rich repeat-containing 15; scRNA-seq, single-cell RNA-sequencing. (TIF) [file pbio.3001805.s008.tif]

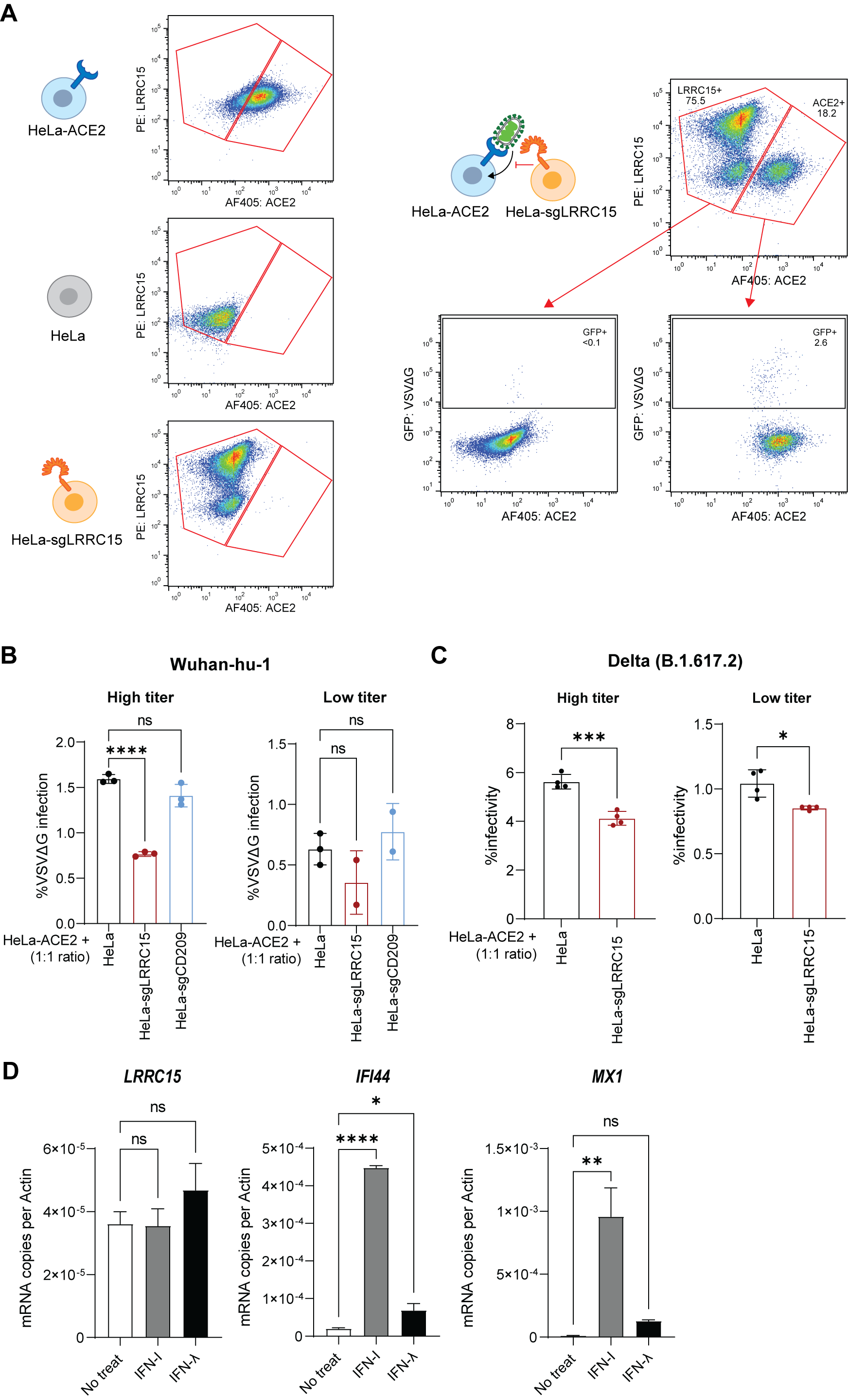

Supplement: S6 Fig — (A) Left, surface ACE2 and LRRC15 expressions of HeLa, HeLa-ACE2, or HeLa-sgLRRC15 cells were measured by flow cytometry. Right, after coculturing HeLa-ACE2 cells with HeLa-sgLRRC15 cells and infecting with VSVΔG-S-SARS2, surface ACE2 and LRRC15 expressions were measured by flow cytometry. GFP expressions were compared in LRRC15+ (HeLa-sgLRRC15) cells and ACE2+ (HeLa-ACE2) cells. (B) HeLa-ACE2 cells were cocultured with HeLa, HeLa-sgLRRC15, or HeLa-sgCD209 cells at 1:1 ratio and infected with VSV pseudovirus–harboring spike of SARS-CoV-2 Wuhan-hu-1 strain at high or low titer. GFP signal was measured at 20 hpi by flow cytometry (n = 4). Representative of 3 independent experiments are shown. (C) Trans-inhibition assay was performed as in (B) with VSV pseudovirus–harboring spike of SARS-CoV-2 Delta variant (B.1.617.2 strain) (n = 4). Representative of 3 independent experiments are shown. (D) A375 cells were treated with Universal IFN-I (100 U/mL) or IFN-λ2 (100 ng/mL) and harvested after 6 h. RNA expressions of LRRC15, IFI44, and MX1 was measured by qPCR and normalized to ACTB. Data represent means ± SD and were analyzed by one-way ANOVA with Dunnett multiple comparisons test (B, D) or unpaired two-tailed t test (C). ns, not significant; *p < 0.05; **p < 0.01; ***p < 0.001; ****p < 0.0001. For underlying data, see S3 Data. ACE2, angiotensin-converting enzyme 2; GFP, green fluorescent protein; hpi, hours postinfection; LRRC15, leucin-rich repeat-containing 15; SARS-CoV-2, Severe Acute Respiratory Syndrome Coronavirus 2; VSV, vesicular stomatitis virus. (TIF) [file pbio.3001805.s009.tif]

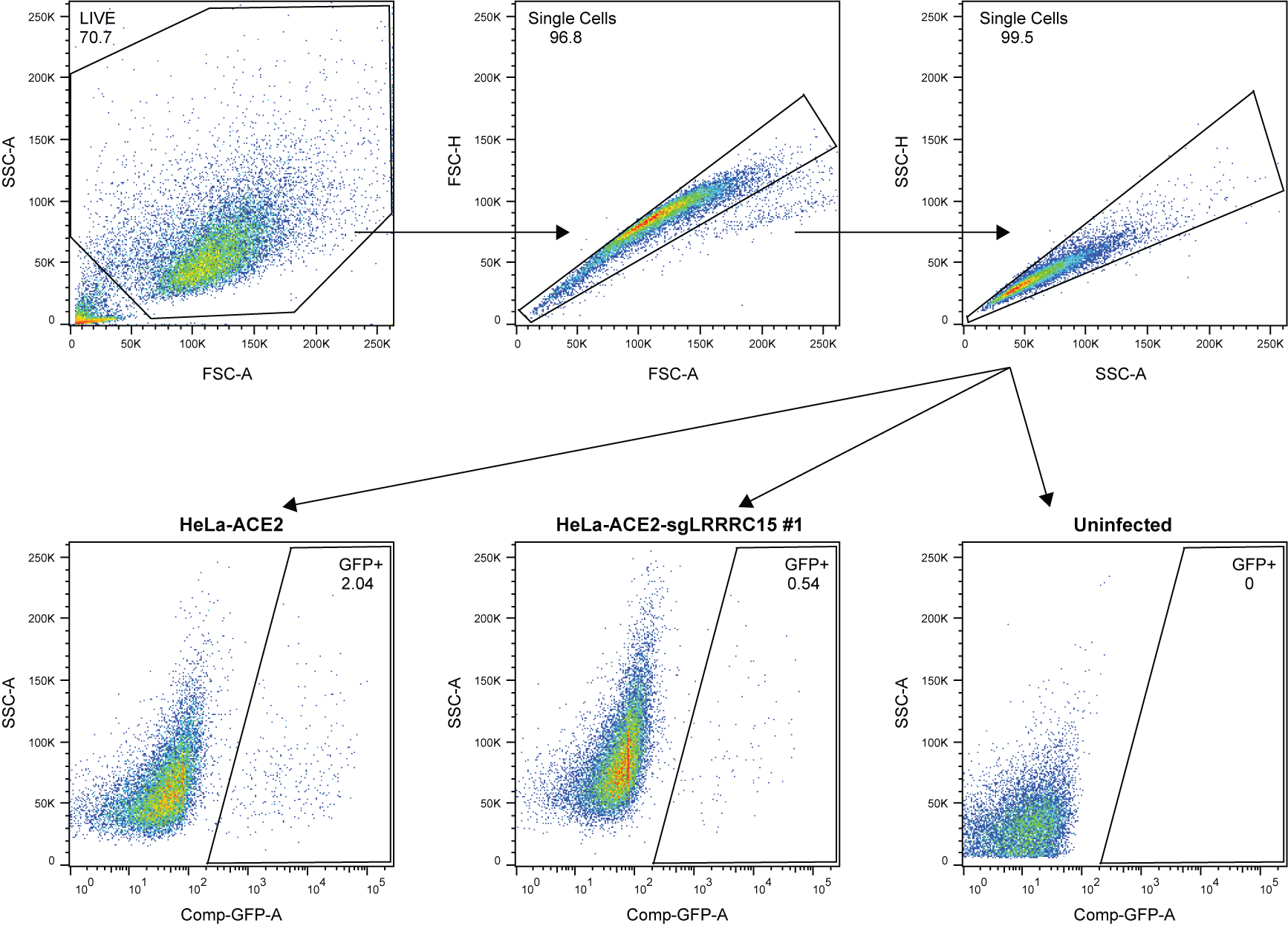

Supplement: S7 Fig — Cells were infected with VSV pseudovirus–harboring spike of SARS-CoV-2, and GFP signal was measured by flow cytometry. Representative datasets for HeLa-ACE2, HeLa-ACE2-sgLRRC15 cells, and uninfected cells are shown. GFP, green fluorescent protein; SARS-CoV-2, Severe Acute Respiratory Syndrome Coronavirus 2; VSV, vesicular stomatitis virus. (TIF) [file pbio.3001805.s010.tif]
