# Supplementary material for: LRRC15 inhibits SARS-CoV-2 cellular entry in trans
Source: PLoS Biol. 2022 Oct 13;20(10):e3001805. doi: 10.1371/journal.pbio.3001805 (PMC9595563; doi:10.1371/journal.pbio.3001805)
Supplement: Attachment — Submitted filename: Point-by-point response_20220803_Resubmission.docx [file pbio.3001805.s011.docx]

Reviewers’ comments:

**Reviewer #1:**
This submission demonstrates that LRRC15 is an integral plasma membrane protein that binds SARS CoV spikes unproductively, in doing so, titrating viruses from ACE2 receptor-mediated virus entry and infection, at least in those conditions where the LRRC15 is overexpressed. The data are solid, although at times not especially striking, and show that the LRRC15 titration effect can be in "cis" (LRRC15 on same cells as ACE2) or in "trans" (LRRC15 not on the same cells as ACE2). The sequestration of virus onto LRRC15 has a modest antiviral effect.  Authors make suggestions about the relevance of LRRC15 in human COVID19, but it remains unclear whether LRRC15 in vivo is sufficiently abundant to absorb viruses and keep them from infecting through ACE2 dependent entry pathways. 
The strengths of the report are with its convincing discovery of LRRC15 as a spike binding protein, the demonstration that LRRC15 binds spike RBDs but does not sterically interfere with ACE2, and with the data that sufficient overexpression of LRRC15 can have some antiviral activity.  The importance of the report comes with the clear identification of a SARS-2 spike binding protein on host cells.  It is important to know about the cells that bind SARS-CoV-2 virus particles.  Of note, there have been several reports of host proteins that attach SARS-2 particles to cells; this submission stands out with strong data that confidently show that LRRC15 binds SARS-2 spikes at low affinity.

There are some concerns with the submission.

1. A central concern is whether the positive results arise only if LRRC15 is vastly overexpressed.  Some activating sgRNAs generate LRRC15 levels exceeding those obtained by direct plasmid based LRRC15 overexpression (Fig. S2 and S3).  At these high overexpression levels, only very modest levels of SARS-2 spikes bind cells (Fig 2).  It takes very high LRRC15 expression to interfere with SARS-2 pseudovirus entry (Fig 3).  Similarly, it takes LRRC15 overexpression to witness trans-inhibition of virus entry (Fig. 6).  It makes sense that vast LRRC15 overexpression is necessary to obtain the cell culture assay readouts, because LRRC15 binds SARS-2 spikes with 200 times lower affinity than ACE2 (for SARS-1 spikes, LRRC15 binding is 1000 times less strong than ACE2).  It may be difficult to compare LRRC15 surface levels in the cell culture experiments to the physiological levels in lung fibroblasts and endothelial cells, but such comparisons would help in determining whether the
findings in the paper have physiological relevance.  Comparison to physiologic LRRC15 levels is recommended.

Author’s response: We examined the expression of LRRC15 in the lung tissue from healthy human donors. The immunofluorescence assay successfully detected LRRC15 protein expression in a subset of cells in the subepithelial layer of alveoli in the human lung specimens, indicating the detectible expression of LRRC15 in normal condition in the lung However, it did not display a clear colocalization either with epithelial (cytokeratin 19), endothelial (CD34), myofibroblast (α-SMA), or lymphatic (Podoplanin) cell marker (*see the figure below*). Although expression of LRRC15 protein was confirmed in the lung of healthy humans, it is difficult to conclude whether LRRC15+ cells are expressed in a subset of fibroblast and/or other cell types with the given data. We decide to leave this topic for future studies and not to include the immunofluorescence staining data in the revised manuscript.

Although it is difficult to directly compare LRRC15 levels between *cultured cell lines and human tissues*, LRRC15 protein was clearly detectable in human lung tissue specimens at comparable signal density by the similar immunofluorescence staining method . Thus, we anticipate that LRRC15 is expressed at levels sufficient to function *in vivo*. Investigating in which specific cell types LRRC15 is expressed will be important for future studies to further clarify its physiological roles.

Importantly, while this paper is in revision, an independent clinical study confirmed the physiological relevance of LRRC15 in human COVID patients in a very recent preprint (Gisby et al., medRxiv, 2022). This multi-omics study identified LRRC15 as a strong predictor of COVID-19 severity in human patients.

Actions: We added a new text in the discussion and cited the preprint publication; “*Importantly, while this article is under review, a multi-omics study identified LRRC15 as a strong predictor of COVID-19 severity in human patients in a preprint.*”

2. A second concern is with the important data in Fig 6; which is aimed at supporting the key conclusion that LRRC15 has “trans-inhibitory” activity.  The spike and LRRC15 signals are very hard to see in Fig 6D and in part this may be because there are no stains for plasma membrane.  Additional PM staining and better imaging of virus-LRRC15 coincidence is recommended.  Also, it is the ratio of cell surface ACE2 to LRRC15 that is relevant, and this could be estimated, as the question is whether it requires hundreds-fold more LRRC15 than ACE2 to effect virus inhibition, given that the virus affinity to LRRC15 is in the 0.1 to 0.2 mM range.

Author’s response: We improved the image data using confocal microscopy and replaced the quantification result in Figure 6. Plasma membrane was visualized by actin co-staining.

To estimate the ratio of ACE2 and LRRC15 level *in vivo*, we compared the number of LRRC15+ and ACE2+ cells using scRNA-seq datasets and found that there are higher numbers of LRRC15+ cells (~3.5-fold in average) than ACE2+ cells. Given that having same number (**Sfig 6B-C**) of or 4-fold more LRRC15+ cells (**Fig 6B-C**) resulted in the significant reduction of viral entry in cell culture system, we anticipate that LRRC15 regulate SARS-CoV-2 infection *in vivo* with this physiological ratio of LRRC15+/ACE2+ cells.

Also, we found that >3-fold more pseudoviruses bind to LRRC15+ cells compared to ACE2+ cells from an attachment assay performed as related to comment #5 of reviewer #2 below (**Fig 4B** in the revised manuscript). Thus, although LRRC15 binds to the spike with ~100-fold lower affinity than ACE2, it can bind to the virus much more strongly possibly with help of multivalency or unknown cofactors, which can be explored in the follow-up studies.

Actions: New confocal microscopic images including actin staining are shown in **Fig 4C** and **6D**. We newly added **SFig 5H** to show the number of LRRC15+ and ACE2+ cells in vivo using the scRNA-seq datasets, and added the corresponding text: “*In lungs from COVID-19 patients [42], the scRNA-seq data revealed that LRRC15-expressing fibroblasts and endothelial cells significantly outnumbered ACE2-expressing epithelial cells (S5H Fig). Specifically, 70% of the samples (14 of 20) showed greater frequencies of LRRC15-expressing fibroblasts and endothelial cells than ACE2-expressing epithelial cells, supporting the relevance of the cell mixing ratios used in Fig 6B-C.*”

3. In virology, the term “receptor” is used for a surface protein that does more than simply bind viruses.  Bona-fide receptors both bind viruses and induce changes in virus that allow for successful virus entry and genome delivery.  By these definitions, LRRC15 is not a receptor.  It is more appropriate to describe LRRC15 as a virus attachment factor (an attachment factor that can interfere with ACE2 receptor-driven entry), not a receptor.

Author’s response: We had used the term “receptor” as LRRC15 binds to the receptor-binding domain of the spike and regulates viral entry, but we agree that it may not be appropriate in this context.

Actions: We edited the manuscript and replaced the term “receptor” with “attachment factor” in the revised manuscript.

Minor points:
1.      Lines 58-60; RBD of SARS-CoV-2 breaks murine host barrier only if there are substitutions such as N501Y; consider rephrasing lines 59-60.

Author’s response: We edited the text to clarify this point.

Actions: We have adjusted the text to “*a single point mutation at the RBD makes it possible to break a barrier of susceptibility of SARS-CoV-2 in murine hosts*” and added a reference for this.

2.      Lines 183-184; unclear what is meant here; is the suggestion that membrane-anchored LRRC15 at high density on cell surfaces allows for multivalent avidity to viruses, while soluble LRRC15 binding to virus relies only on affinity, not avidity?  Also, lines 185-187; data do not appear to allow any conclusions about LRRC15 “restricting the internalization” of virions.

Author’s response: We apologize for the confusion. In related to comment #4 from reviewer #3 below, we think the partial suppression of pseudovirus infection by LRRC15 is largely due to its low binding affinity compared to ACE2. As we did not show whether multivalency or membrane localization of LRRC15 does affect its function, we decided to delete the sentence.

We added a result of an internalization assay to show whether LRRC15 does affect the internalization of pseudoviruses. Please refer to comment #6 of reviewer #2 below for further detail.

Actions: We replaced the text of lines 183-184 in the previous manuscript with “*The difference in blocking efficacy is likely the consequence of different spike binding affinity of LRRC15 and ACE2.*” We added new data and corresponding text for the internalization assay; a representative image in **Fig 4C** and quantification data in **Fig 4D**.

3.      Fig. S2D; Kd for SARS-1 RBD to ACE2 is 0.16 nM, higher affinity than SARS-2 RBD.  This is not consistent with many reports that SARS-2 RBD has the higher affinity for ACE2.

Author’s response: We measured SARS-CoV-2 RBD-ACE2 affinity in the same ELISA experiment, with ACE2-His coated on the bottom and recombinant RBD protein added on top. In this setting, Kd for SARS-2 RBD to ACE2 was 0.059 nM, indicating SARS-2 RBD has higher affinity to ACE2 compared to SARS-1 RBD.

Actions: We replaced the data (shown in **Fig S2E** in the revised manuscript) to show the binding affinity of SARS-CoV and SARS-CoV-2 RBD to ACE2.

4.      Line 338; not clear whether the trans-inhibition in HeLa cell context “indicates that the antiviral effect of LRRC15 can be broad in a physiological context”.  Consider tempering conclusions here.

Author’s response: We agree that the previous description was unclear. The revised discussion was added.

Actions: We added a revised sentence, “*It suggests the antiviral effect of LRRC15 can be broad functional in a broader physiological context which does not require cell-intrinsic restriction in SARS-CoV-2 susceptible cells in the lung.*”

**Reviewer #2 (Teunis BH Geijtenbeek):**

Here the authors have identified LRRC15 as an attachment receptor for SARS-CoV-2 that does not by itself mediate entry. Co-expression with ACE2 on cell-lines strongly suggest that LRRC15 suppresses ACE2-mediated infection of SARS-CoV-1. Using public data sets they show that LRRC15 is not expressed by ACE2-positive cells but rather by lymphatic endothelial cells. They have identified pathological fibroblasts as a major cell type expressing LRRC15 which are present in lungs of deceased SARS-CoV-2 patients. They suggest that LRRC15 has an antiviral function as high expression correlates with low viral load in deceased patients. Finally they show that LRRC15 can inhibit virus infection in trans.
The identification of LRRC15 as an attachment receptor is interesting and the inhibitory in trans function is novel but lacks proper controls to convincingly show it is a novel function. The authors attempt to find a physiological role for LRRC15 in data sets from lungs of deceased SARS-COV-2 Patients but the conclusions are overstated as no real correlation can be made without extensive testing. All patient data are based on transcriptomics/RNA seq but protein expression is not investigated. Moreover, in order to exert the putative inhibitor function the cells need to be localized next to each other. The localization of the cells compared to ACE2 cells needs to be investigated. Finally, everything has been done with pseudotyped viruses and cell-lines, which lowers relevance. SARS-CoV-2 isolates need to be used in binding and infection experiments to understand whether LRRC15 is able to inhibit actual virus. Coronaviruses express high levels of Spike protein on membrane,
whereas Spike on pseudoviruses will be much less and this has a major impact on the findings.

Major concerns
1. Only pseudotyped virus is used and the major experiments need to be performed with SARS-CoV-2 isolates. This is essential as the Spike proteins are much higher on actual virus than pseudovirus.

Author’s response: We used a replication-competent isolate of live SARS-CoV-2 with a fluorescent reporter mNeonGreen (icSARS-CoV-2-mNG) and measured the infectivity in HeLa-ACE2 and Huh7.5 cells where LRRC15 expression was induced using the CRISPR activation. Decreased infection of SARS-CoV-2 in LRRC15-induced cells was confirmed in both cell lines.

Actions: We added new figures (**Fig 3D-E**) to demonstrate LRRC15 inhibits the infection of live SARS-CoV-2 in HeLa-ACE2 and Huh7.5 cells.

2. The LRRC15 binding to S protein is very weak (Fig 2). These data should be quantified. Here both SARS-CoV-2 and pseudotyped virus can be used to measure binding as multivalency might increase interaction (similar as has been done for Fig 4).

Author’s response: We included the mean fluorescence value for Spike S1-Fc or NTD/RBD-Fc binding in Fig 2A and 2C. We employed a His-tagged, full length spike protein capable of forming trimer to examine whether trimerization of spike enhances LRRC15-spike binding. The binding assay was conducted using different concentrations of spike proteins and LRRC15-spike binding was observed but it was much weaker than ACE2-spike binding.

We tried to measure binding of SARS-CoV-2 pseudovirus to LRRC15-expressing cells by flow, but unfortunately, virus binding could not be detected by using anti-spike antibody. Instead we measured the virus binding to ACE2-negative/LRRC15-positive cells by quantifying viral genome using RT-qPCR, and showed these cells bind with the pseudovirus stronger than ACE2 single-positive cells. Please see comment #5 below for further detail.

Actions: We have added mean fluorescence intensity for S1 or NTD/RBD-Fc binding for each cell line in histograms in **Fig 2A/C**. Histograms showing full-length spike binding to LRRC15+ or ACE2+ cells were added **Sfig 2C**. Virus attachment assay shown in **Fig 4B** was replaced with a new data to include LRRC15 single-positive cells.

3. Induction of LRRC15 on ACE2 cells inhibits pseudovirus infection (Fig 3). The data are relative and actual infection data for all relative data should be shown to understand the impact on infection.

Author’s response: All infectivity data were replaced with actual infection rate. The infection rate for VSV-∆G pseudoviruses are between 0.5~5% in HeLa-ACE2 cells depending on the strain of SARS-CoV-2 spike. The infection rate is low since replication-incompetent pseudovirus was used, which is consistent with the previous reports (Capcha et al., Front Cardio Med; PMID: 33521067). It was reported that the lack of entry cofactors, like TMPRSS2 in HeLa cells might lead to a low infection rate compared to other highly susceptible cell lines. In each experiment, we confirmed negligible reporter signal (i.e., <0.1% infection rate) in mock-infected cells and analyzed >10,000 cells to prevent the detection of background noise signal.

Actions: All of the pseudovirus infection data in **Fig 3** and **Sfig 3** were replaced to present actual infection rates.

4. There is a small effect of LRRC15 induction but not overexpression on ACE2 expression and this should be quantified to understand relevance. What would happen if LRRC15 was induced on epithelial cells or CALU-3 cells that have endogenous ACE2 expression?

Author’s response: We analyzed the mean fluorescent intensities for surface ACE2 level in each HeLa-ACE2 cell line. ACE2 level showed slight decrease or increase in different cell lines, but it did not correlate with the infection phenotype. For example, surface ACE2 was marginally decreased by sgLRRC15 #1 and not by sgLRRC15 #2, while both guide RNAs equally suppressed viral entry. We also measured surface ACE2 expression in LRRC15-induced Huh7.5 cells which naturally expresses endogenous ACE2. ACE2 level, which was much lower than HeLa-ACE2 cells, was rather slightly increased by sgLRRC15 #1 and 2. Entry of the pseudotyped virus or live SARS-CoV-2 infection was efficiently suppressed in the Huh7.5 cells. Thus, we conclude that LRRC15 restricts the spike-mediated entry independent of the surface level of ACE2.

Actions: We added a figure showing surface ACE2 level in Huh7.5 cell lines in **Sfig 4** and included MFI for surface ACE2 for each cell line in **Fig 4A**. Corresponding text was changed as follows: “*The level of ACE2 surface expression was unaltered or marginally decreased by sgRNA-mediated gene induction in HeLa-ACE2 cells and was slightly increased in LRRC15-induced Huh7.5 cells (Fig 4A, S4A Fig). Although surface ACE2 expression is slightly different, they all showed the decreased entry of SARS-CoV-2 pseudotyped virus and authentic virus, indicating the inhibitory effect of LRRC15 does not require the regulation of surface ACE2 level.*”

5. Fig 4B. The authors state that virus is sequestered by LRRC15 on ACE2-positive cells but here they should also show ACE2-negative cells as it is possible that the increase is due to LRRC15 and virus binding.

Author’s response: We thank the reviewer for raising this point. We included ACE2-negative/LRRC15-positive cells (HeLa-sgLRRC15 #1) in the attachment assay, shown in the new **Fig 4B**. It showed a strong binding with SARS-CoV-2 pseudotyped virus (even stronger than ACE2 single-positive cells), which supports our model of LRRC15 sequestrating virus from entering ACE2+ cells *in trans*. The increase of virus-binding on ACE2+LRRC15+ cells is likely due to the LRRC15-virus binding.

Actions: We replaced **Fig 4B** with a new figure including HeLa-sgLRRC15 #1 cells and edited the corresponding text, “*As expected, SARS-CoV-2 spike-pseudotyped viruses bound to ACE2-expressing cells and the binding was not altered by inducing a control gene, CD45. Importantly, viruses were highly accumulated in LRRC15-induced cells, both in the absence and presence of ACE2 (i.e., ~3-fold increases in viral copies) compared to their controls (Fig 4B).*”

6. Based on these data (Fig 4B) the authors suggest that LRRC15 sequesters virus on cell-surface and prevents internalization (line 174). But this cannot be concluded from an experiment performed on ice. The authors need to show that the virus in presence of LRRC15/ACE2 is not internalized compared to ACE2 alone. For example by trypsin removal of extracellular bound virus and pulse chase experiments or tracking by immunostainings.

Author’s response: We conducted internalization assay as suggested. Cells were incubated with spike-pseudotyped viruses for 1 hr on ice, washed thoroughly with cold media, and then incubated for additional 1 hr at 37°C to allow internalization. Spikes were detected with immunostaining. We found that few spikes were detected after 1 hr internalization in ACE2 only cells as viruses successfully entered the cells. On the contrary, significant number of spikes were detected even after 1 hr in the presence of LRRC15, indicating LRRC15 inhibits the virus from entering the cells. We also included DC-SIGN induced cells as suggested.

Actions: We added a new data for the internalization assay; a representative image in **Fig 4C** and a quantification data in **Fig 4D**. Corresponding text was added, “*Immunofluorescence staining of spike proteins confirmed the enhanced binding of pseudoviruses to LRRC15- but not to DC-SIGN-induced cells (Fig 4C-D, S4B Fig). While few spikes were detected in HeLa-ACE2 cells after one hour of internalization, significant number of spikes were detected in LRRC15-induced cells, indicating the internalization of viruses is inhibited by LRRC15 expression (Fig 4D).*”

7. The possible association of LRRC15 expression with deceased SARS-CoV-2 patients (Sfig 5, Fig5) is very suggestive and not convincing. The pathophysiological role is highly speculative and not relevant as it is unclear how expression of lRRC15 is regulated. Further experiments would be required such as co-localization of LRRC15 positive cells and epithelial cells, and actual infection of epithelial cells and expression of LRRC15 (Fig 5E). Without more concrete data it is very difficult to convince that LRRC15 has an antiviral function *in vivo*. The low virus load could be due to many different processes as also shown by the high amount of differently expressed genes and as such it is an overstatement to state that LRRC15 might contribute to the observed decrease of virus load.

Author’s response: As related to comment #1 from reviewer #1 above, we tried to detect LRRC15 proteins in human lung tissue samples by immunostaining. We found LRRC15 is clearly present in a subset of cells in the lung, while ACE2 protein was detected in cells different from, but located near LRRC15+ cells.

As described in the comment #1 of the reviewer #1, while this paper is in revision, an independent clinical study confirmed the physiological relevance of LRRC15 in human COVID patients in a very recent preprint (Gisby et al., medRxiv, 2022). This multi-omics study identified LRRC15 as a strong predictor of COVID-19 severity in human patients.

Actions: We added a new discussion and cited the preprint; “*Importantly, while this article is under review, a multi-omics study identified LRRC15 as a strong predictor of COVID-19 severity in human patients in a preprint.*”

8. Fig 6. infectivity is very low 2% or 0.3%. Why is the infection so low and this makes the effect although significant also less relevant. Moreover, controls need to be included to show that the co-culture with just any attachment receptor does not prevent infection. So ACE2-positive cells should be co-cultured with DC-SIGN-positive cells (which have shown to enhance infection in trans/cis). Is a ratio of 1:4 physiologically relevant? It is likely that LRCC15 sequesters virus and thereby lowers virus concentration in the medium leading to lower infection so this needs to be carefully controlled.

Author’s response: The trans-inhibition assay shown in **Fig 6** employed a co-culture system where 80% of cells are ACE2-negative, resulting in a much lower infection rate. As we did for infection assays shown in **Fig 3**, we analyzed sufficient number of cells to include at least >100 infected cells in each replicate. We included DC-SIGN-induced cells as suggested and confirmed the trans-inhibitory function is specific to LRRC15. Co-culture the cells with DC-SIGN-positive cells made a slight but statistically not significant increase of viral infection.

In terms of the co-culture ratio, we analyzed the scRNA datasets and found that there are more LRRC15+ cells than ACE2+ cells in vivo, so we think having more LRRC15+ cells for coculture experiment is relevant. Although the ratio of 1:4 is arbitrarily selected, LRRC15 could also suppress viral entry when 1:1 ratio was used in **SFig 6B-C**, so LRRC15 may be able to regulate ACE2-mediated entry in trans, when at least same or more LRRC15+ cells are present compared to ACE2+ cells.

Actions: We replaced **Fig 6B** with a new data including co-culturing with DC-SIGN-positive cells and added the corresponding text, “*co-culturing with DC-SIGN-expressing cells did not induce the reduction, confirming the specificity of inhibitory function of LRRC15*”.

9. The colocalization data are very unclear and it is difficult to assess co-localization. significance should be denoted in Fig 6E after quantification.

Author’s response: We improved the image quality by using confocal microscopy, and higher number of spike proteins were detected. Spike speckles in LRRC15- and LRRC15+ cells were counted and they showed a statistically significant increase compared to cells co-cultured with naïve HeLa cells.

Actions: **Fig 6D** was replaced with new images obtained with a confocal microscope and the quantification data in **Fig 6E** was replaced to show the statistical significance.

**Reviewer #3:**

In this manuscript, Dr. Wilen and Lee's groups have used a genome-wide CRISPR activation screening to determine novel SARS-CoV-2 spike binding partners. They used ACE2 as a positive control for their screens, and identified LRRC15 as a new interaction partner specifically for the spike protein of SARS2/various SARS2 variants, as well as SARS1. A serial of experiments were also performed to verify this interaction, and the binding epitope was mapped to RBD, although with much weaker affinity compared to ACE2 (~100 fold less by ELISA assay). Functionally, LRRC15 itself does not permit viral entry as indicated by VSV-G SARS-2 spike pseudotyped virus infection experiments. However, LRRC15 provided in cis (on the same ACE2 positive cells, such as A375 melanoma or Hela cells) or trans (LRRC15+ cells plus ACE2+ cells) significantly inhibited SARS-2 spike pseudovirus infection. Mechanistically, the authors provided some evidence that LRRC15 may capture SARS-2 spike
pseudovirus and potentially render viron sequestration. Given the fact that LRRC15 showed distinct expression pattern compared to ACE2, the author proposed that LRRC15 provided in trans on SARS-2 non-permissive fibroblasts may protect ACE2 permissive cells against viral infection, and LRRC15 is an inhibitory molecule for SARS2 viral entry by directly interacting with the spike protein. 
Overall, this study contains novel findings and is quite interesting to the field. The paper was also well-written. However, there are several technical concerns and a lack of detail mechanisms which I see for potential improvement.

1. Since this is a genome-wide CRISPRa screening, did the authors identify any other molecules besides LRRC15 from this screen? There are around 10 other SARS-2 binding partners (C-type lectins, AXL, Siglec, Kremen-1 etc,) being identified through various screening platforms, the comparison of CRISPRa with other methods will provide important technical insights to the field. Moreover, the authors may discuss the advantage and disadvantages of CRISPRa screen vs others in this specific experimental setting.

Author’s response:

We checked whether other SARS-CoV-2 spike-binding factors are enriched in our CRISPRa screen but none of them showed a significant enrichment in both two biological replicates. This may be due to their relatively weak spike-binding affinities (Hoffmann et al., EMBO, 2021) or dependent on cell types expressing different cofactors. Different screening methods may also account for the discrepancy. Most of the SARS2 spike attachment factors were identified using cDNA array (Lu et al., Immunity, 2021; Gu et al., Cell Research, 2021). Compared to cDNA expression system, CRISPRa induces a robust gene expression, allowing the identification of a low affinity targets. Also, using four different guide RNAs per gene prevents false-positive discovery. Our CRISPRa screen used a focused library targeting ~6000 genes enabling a higher sequencing depth and better screening resolution and reproducibility compared to genome-wide libraries targeting >20,000 genes.

Actions: We added **SFig 1B** in the edited manuscript to show other spike-binding factors in our CRISPRa screen. The text was added. “*Other reported spike attachment factors were not significantly enriched in our screen [24, 26, 27] (S1B Fig). This discrepancy might be from relatively weak spike-binding affinities of previously identified attachment factors [24, 27, 28] or dependent on cell types expressing different cofactors.*”

2. In figure 2, the LRRC15 binding data look quite weak in general. However, it seems specific although the Kd of this interaction is ~200 fold lower based the direct protein-protein interaction measurement by ELISA. Other than RBD, does CTD or S2 contribute to LRRC15 binding? Moreover, the difference between Spike binding to ACE2 and LRRC15 seems much smaller in Delta or Iota variants, the authors may need to discuss whether there is any meaning interpretation on these interesting findings.

Author’s response: We measured the binding of full-length spike protein to LRRC15-expressing cells by flow, which was still weaker than ACE2+ cells. Also, RBD-Fc showed much stronger binding to LRRC15+ cells compared to S1-Fc or full-length spike protein. Although we did not measure direct binding of LRRC15 to CTD or S2, it is not likely they contribute to LRRC15 interaction.

We agree that different LRRC15-binding affinities of SARS-CoV-2 variants are of interest. Although LRRC15 reduced the entry of pseudoviruses of all tested variants at a similar fold in HeLa-ACE2 cells, different binding affinity may alter the phenotype in different settings like naturally susceptible cells or infection in the lung, which will be explored further in future studies.

Actions: We added the additional interpretation text in the result, “*Although LRRC15 reduced the entry of pseudotyped viruses of all tested variants at a similar fold in HeLa-ACE2 cells, different binding affinity may alter the phenotype in different settings like naturally susceptible cells or infection in the lung.*”

3. Using SARS-2 spike VSV-G pseudovirus, the authors found LRRC15 itself does not elicit pseudovirus infection, which is different from VSV-G spike pseudovirus. Coexpression of both LRRC15 and ACE2 significantly inhibited SARS2 pseudovirus infection. This data is interesting, however, requires delicate validation with active SARS-2 virus. The infection mechanism of VSV-G pseudovirus has been found to be quite different from the real SARS-2 virus (PMCID: PMC8106883). For instance, DC-SIGN/L-SIGN can elicit pseudovirus infection, but these molecules did not elicit active replication of SARS2 virus. The authors may also need to include DC-SIGN as a control to show whether their pseudovirus can infect receptors other than ACE2, and whether LRRC15 in cis could inhibit ACE2 dependent SARS2 real virus infection.

Author’s response: As related to comment #1 from reviewer #2 above, we confirmed LRRC15 function is also applicable to live SARS-CoV-2 virus (**Fig 3D-E**). We induced DC-SIGN (CD209) in HeLa cells and they were not permissive to VSV-∆G-SARS2 S, indicating the pseudovirus infection is exclusively dependent on ACE2 expression.

Actions: We added new figures (**Fig 3D-E**) and corresponding text in results section to demonstrate LRRC15 inhibits the infection of live SARS-CoV-2 in HeLa-ACE2 and Huh7.5 cells. We also included HeLa-sgCD209 cells for pseudovirus infection assay shown in **SFig 3B**.

4. Mechanistically, the authors did not find LRRC15 can modulate ACE2. In figure 4B, LRRC15 CRISPRa induction on the top of ACE2 was found to increase viron binding. However, a LRRC15 only control is required for this experiment. The authors found LRRC15 fusion protein can partially block pseudovirus binding (Fig 4C), in a much weaker fashion than ACE2, which could be largely attributed to the weak affinity of LRRC15 to spike RBD compared to ACE2. Therefore, the authors reasoned that “the inhibitory function of LRRC15 may require localization to the cellular membrane and/or the cytosolic domain of LRRC15 for its functional efficiency”, which is not convincing in this case. Moreover, LRRC15 fusion could not block ACE2-spike binding, which is not enough to conclude that LRRC15 could not compete with ACE2, particularly in cis. Moreover, does ACE2 fusion protein block LRRC15/RBD binding? This is important and will provide important information that whether LRRC15 may share some
similar epitopes with ACE2 within RBD.

Author’s response: As related to comment #5 from reviewer #2 above, we included LRRC15 only cells control for attachment assay.

We agree that different efficacy of ACE2 or LRRC15 protein to block pseudovirus infection (shown in **Fig 4E** in the edited manuscript) is due to their different binding affinity, and it is hard to conclude membrane localization of LRRC15 matters in this context.

We measured LRRC15-spike binding in the presence of excess ACE2 recombinant protein, which was not altered even with high concentration of ACE2. Since both ACE2 and LRRC15 did not block spike-binding of each other, this result suggests their non-overlapping epitopes on the RBD.

Actions: We replaced **Fig 4B** with a new figure to include pseudovirus binding to LRRC15 only cells. We replaced the text describing **Fig 4E** with “*The difference in blocking efficacy is likely the consequence of different spike binding affinity of LRRC15 and ACE2.*”

We have added **Fig 4G** to show ACE2 does not block LRRC15-spike binding and added text “*Conversely, spike-LRRC15 interaction was not affected by excess ACE2, demonstrating LRRC15 and ACE2 does not share a same binding epitope within the RBD (Fig 4G).*”

5. The authors only showed that LRRC15 enhanced pseudovirus binding in the presence of ACE2. Does LRRC15 elicit direct virus endocytosis and degradation (no replication), in the condition with or without ACE2? This is a valid hypothesis that requires detail characterization, particularly in the absence of ACE2.

Author’s response: As related to comment #6 from reviewer #2 above, we performed an internalization assay. When LRRC15 was induced in ACE2+ cells (**Fig 4C-D** in the edited manuscript), spike proteins of the pseudoviruses remained attached to the cells after 1 hr of internalization which was sufficient for most viruses to enter the cells without LRRC15. Similarly, a number of spikes were detected on the surface of LRRC15 only cells while the co-cultured LRRC15- allowed the internalization of most spikes as shown in **Fig 6D-E**. Thus, we hypothesize that LRRC15 sequestrates the virus on the surface and prevents the endocytosis of the virus.

Actions: We added a new data for the internalization assay; a representative image in **Fig 4C** and a quantification data in **Fig 4D**. Corresponding text was added to the results section.

6. The LRRC15 expression data is rather interesting, which indicates LRRC15 expression in ACE2 negative cells. Importantly, LRRC15 provided in trans also inhibited SARS2 pseudovirus infection. Again, this experiments require further validation using real virus. Given the fact that the C-type lectins in trans can enhance SARS2 infection, the authors may need to include DC-SIGN as a control for this experiment, and measure the endocytosis of virus by LRRC15/DC-SIGN/ACE2, to pinpoint specific mechanisms.

Author’s response: Although we confirmed LRRC15 suppresses live SARS-CoV-2 infection in cis, we could not validate LRRC15 function *in trans* using live virus due to technical limitations with the BSL3 facility.

As related to comment #8 from reviewer #2 above, we included DC-SIGN+ cells as a control for co-culture experiment and found no significant increase of viral entry in ACE2+ cells. The trans-infection assay conducted by Lempp et al. employed a sequential incubation of DC-SIGN+ cells first with virus and then with ACE2+ cells to show DC-SIGN binds to SARS-CoV-2 strong enough to transfer the virus to ACE2+ cells. On the other hand, we incubate ACE2+ and DC-SIGN+ cells with the virus at the same time and DC-SIGN may not enhance ACE2-virus binding further in this experimental setting.

Actions: We replaced **Fig 6B** with a new data including co-culturing with DC-SIGN-positive cells and added the corresponding text, “*co-culturing with DC-SIGN-expressing cells did not induce the reduction, confirming the specificity of inhibitory function of LRRC15.*”

7. TMPRSS2 has been suggested to be critical to SARS2 infection, what is the role of LRRC15 in the presence of this protease?

Author’s response: We treated cells with the TMPRSS2 inhibitor camostat mesylate to examine whether TMPRSS2 and LRRC15 are related. As HeLa-ACE2 cells do not express TMPRSS2, we used HEK293T-ACE2/TMPRSS2 cells which are sensitive to camostat treatment and overexpressed LRRC15 in these cells. Pseudovirus entry was decreased by LRRC15 expression only in camostat-treated but not in DMSO-treated cells, suggesting LRRC15 cannot regulate SARS-CoV-2 entry in the presence of TMPRSS2. The virus may overcome LRRC15 when the entry process is facilitated by TMRPSS2-mediated spike cleavage. A detailed examination of whether LRRC15 regulates the endocytic pathway-mediated viral entry will be an important question for the follow-up studies.

Actions: We added **SFig 3H-I** to show that LRRC15 suppresses SARS-CoV-2 entry in camostat-treated 293T-ACE2/TMPRSS2 cells and added text to the results section, “*Cellular serine protease TMPRSS2 facilitates viral entry through the plasma membrane route during SARS-CoV-2 infection [9]. Treatment of TMPRSS2 inhibitor camostat in HeLa-ACE2 cells did not alter the pseudoviral entry as they do not express endogenous TMPRSS2 [38], and the inhibitory effect of LRRC15 was conserved in camostat-treated cells (S3H Fig). We employed the HEK293T cells ectopically expressing ACE2 and TMPRSS2 (293T-ACE2/TMPRSS2) to model the entry pathway through the plasma membrane fusion. In the 293T-ACD2/TMPRSS2 cells, treatment of camostat showed a reduced entry of the SARS-CoV-2 pseudovirus. In 293T-ACE2/TMPRSS2 cells, LRRC15 suppressed viral entry only upon camostat-treatment but not with control-treatment (S3I Fig), implying the endosomal pathway of SARS-CoV-2 entry is sensitive to the entry restriction by LRRC15 over plasma membrane fusion pathway.*”
