# Supplementary material for: LRRC15 inhibits SARS-CoV-2 cellular entry in trans
Source: PLoS Biol. 2022 Oct 13;20(10):e3001805. doi: 10.1371/journal.pbio.3001805 (PMC9595563; doi:10.1371/journal.pbio.3001805)
Supplement: Attachment — Submitted filename: Point-by-point response_20220819_SLedit.docx [file pbio.3001805.s012.docx]

Reviewer remarks:

**Reviewer #1:**
This revised submission is improved. The essential conclusions of the submission are now more adequately defended with new findings. The findings that LRRC15 suppresses authentic SARS-CoV-2 are valuable. The article is a significant contribution. Notably some of the findings are largely concordant with independently obtained results from other labs who had identified LRRC15 as a virus attachment factor.

Remaining Comment:

Can the authors please point out the IFA signals for spike in Figs 4C and 6D?  The authors claim that VSV-SPIKE pseudo viruses bind LRRC15+ cells in Fig 4C but this reviewer is not confident of the signals deemed positive vs those considered negative. In Fig 4C there seem to be no positive spike signals on LRRC15 cells but they seem to be present in Fig 6D. Again, it would be helpful to point out what green fluorescence is and is not deemed evidential pseudo virus binding with some arrows.

Author’s response: We added white arrowheads to point out the green fluorescence signal we considered positive for spike proteins in Figure 4C and 6D. In each experiment, mock-infection control was included to specify the green fluorescence signal and fluorescence signals that are substantially bigger and brighter than the negative control samples (*data not shown*).

Actions: We added white arrowheads to indicate specific fluorescence signals for spike proteins in **Figure 4C** and **6D**.

**Reviewer #2 (Teunis BH Geijtenbeek):**
The authors have addressed the concerns with new data and clear explanations. The manuscript has improved and I have no other concerns.

Author’s response: Thank you for constructive feedbacks.

**Reviewer #3:**
The authors have reasonably answered my concerns. Some minor issues:
1. The authors commented that LRRC15 sequestrates the virus on the surface and prevents the endocytosis of the virus, which is lack of clear evidence. It remains important to test whether LRRC15 in cis or in trans can mediate virus endocytosis, in a way potentially important to reduce virus titer. The identification of virus on the cell surface is not enough to dissect this question. More experiments, for example, whether LRRC15 effect is lost in 4 degree vs 37 degree, and/or the inclusion of endocytosis inhibitors, are necessary.

Author’s response: We agree that the current data may not provide direct evidence of modulation of viral endocytosis or internalization. We revised the corresponding text as the reviewers and the editor suggested.

Actions: We replaced the text for **Fig 4D** as follows: *“implying that LRRC15 may inhibit the internalization of the virus (Fig 4D).”*

2. The authors may need to include some discussion on the limitation of this study--such as the inhibitory effect of LRRC15 on virus infection mediated by ACE2 could be conditional, particularly in physiological conditions.

Author’s response: We added additional description in the discussion to state the limitation of this study related to the potential contribution of LRRC15 and TMPRSS2-dependence in the physiological context.

Actions: We added following text in the discussion section: *“As we did not see an effective restriction activity of LRRC15 on cells overexpressing TMPRSS2 (S3I Fig), it is possible that LRRC15 exhibits differential restriction activities depending on entry pathways of SARS-CoV-2. Further investigation for the differential sensitivity will be necessary to determine the dependence of enzymatic entry co-factors including TMPRSS2, cathepsins or metalloproteinases in different cell types with their endogenous expression.”* and *“although such inhibitory function may vary, depending on relative distribution and expression of LRRC15 and ACE2 in vivo”.*

3. The explanation on the data related to TMPRSS2 is still not convincing. The authors found LRRC15 suppressed viral entry only upon camostat-treatment, but not with control treatment, which may indicate the role of LRRC15 can be dependent on, at least in the case of TMPRSS2 overexpression. Given the fact that TMPRSS2 is important for ACE2 priming, the author's statement that "In 293TACE2/TMPRSS2 cells, LRRC15 suppressed viral entry only upon camostat-treatment but not with control-treatment (S3I Fig), implying the endosomal pathway of SARS-CoV-2 entry is sensitive to the entry restriction by LRRC15 over plasma membrane fusion pathway" may be not accurate.

Author’s response: We agree that our conclusion of the camostat experiment could be not accurate. We revised the corresponding text in the results section and the discussion about this as related to comment #2 above.

Actions: We added new sentences in the discussion section to address this point and replaced the text for S3I Fig as follows: *“implying that TMPRSS2-independent SARS-CoV-2 entry is more sensitive to the entry restriction by LRRC15.”*
